# Neural dynamics during emotional video engagement relate to anxiety

**DOI:** 10.3389/fnhum.2022.993606

**Published:** 2022-11-11

**Authors:** Jason Nan, Pragathi P. Balasubramani, Dhakshin Ramanathan, Jyoti Mishra

**Affiliations:** ^1^Neural Engineering and Translation Labs, Department of Psychiatry, University of California, San Diego, San Diego, CA, United States; ^2^Department of Bioengineering, University of California, San Diego, San Diego, CA, United States; ^3^Department of Cognitive Science, Indian Institute of Technology Kanpur, Kanpur, India; ^4^Department of Mental Health, VA San Diego Medical Center, San Diego, CA, United States; ^5^Center of Excellence for Stress and Mental Health, VA San Diego Medical Center, San Diego, CA, United States

**Keywords:** EEG, inter-subject correlation, anxiety, mood, emotion, parietal

## Abstract

Inter-subject correlations (ISCs) of physiological data can reveal common stimulus-driven processing across subjects. ISC has been applied to passive video viewing in small samples to measure common engagement and emotional processing. Here, in a large sample study of healthy adults (*N* = 163) who watched an emotional film (The Lion Cage by Charlie Chaplin), we recorded electroencephalography (EEG) across participants and measured ISC in theta, alpha and beta frequency bands. Peak ISC on the emotionally engaging video was observed three-quarters into the film clip, during a time period which potentially elicited a positive emotion of relief. Peak ISC in all frequency bands was focused over centro-parietal electrodes localizing to superior parietal cortex. ISC in both alpha and beta frequencies had a significant inverse relationship with anxiety symptoms. Our study suggests that ISC measured during continuous non-event-locked passive viewing may serve as a useful marker for anxious mood.

## Introduction

Humans, as social creatures, tend to mirror the emotions of others they see, whether in person or digitally. Witnessing a person walk across a tightrope might induce most viewers to feel anxious and stressed, while a cute video of a cat might be heartwarming (Uhrig et al., 2016). Recent studies have quantified the neural correlates of mirroring or “sync up” with others by calculating inter-subject correlation (ISC) of neuroimaging and electrophysiological data analyzed on naturalistic stimuli such as films and movie clips. In essence, ISC is a useful neural marker in answering the question “How alike is one subject to the rest of the group?”. The first major study in this field measured blood-oxygen-level-dependent (BOLD) activity as subjects viewed sections of a movie. High levels of correlated brain activity between subjects were observed in areas beyond basic sensory processing regions, including the limbic system and superior temporal cortex (Hasson et al., 2004). This suggested that there are common, temporally similar patterns of activity in higher order associative processing regions. Follow up studies (Jääskeläinen et al., 2008; Nummenmaa et al., 2012; Di and Biswal, 2020), using functional magnetic resonance imaging (fMRI) or functional near infrared spectroscopy (fNIRS), have corroborated Hasson’s earlier findings, showing ISC in a number of brain regions beyond basic sensory cortex. Additional studies have shown this effect is likely related to emotional processing or engagement and not strictly external stimulus dependent, as ISC can be elicited with narrative content in either written or audio form (Regev et al., 2013; Wang and He, 2014).

The BOLD signal measured in most of the studies noted above is a proxy for actual neural activity (frequency band power) and is expensive to measure. Electroencephalography (EEG), which is a low-cost method that directly reflects physiological activity of neural populations, has also been used to measure ISC (Dmochowski et al., 2012, 2014). Such studies have corroborated those noted above, demonstrating that ISC in EEG signals is greater during periods of high engagement and arousal (Dmochowski et al., 2012; Cohen et al., 2017, 2018). For example, subjects hearing the same song in a repetitive manner showed lower ISC compared to a remix of the same song that had less predictable attributes, which would cause subjects to be more attentive (Dauer et al., 2021). Similarly, when subjects were presented an audiovisual stimulus, lower ISC was observed when they were distracted with mental math (Ki et al., 2016). One additional benefit of EEG is the high temporal resolution which allows frequency band filtering of specific brain activity. Theta band activity is known to increase in response to facial stimuli that occur during naturalistic viewing (Busch et al., 2009; Dravida et al., 2019). Beta oscillations have been associated with attentiveness (Posada-Quintero et al., 2019), while in other contexts, alpha and beta oscillations have been related to anxiety and stress (Knyazev et al., 2004; Price and Budzynshi, 2009; Abhang et al., 2016). In fact, a study has already noted that ISC during emotional states does show distinct differences across frequency bands, though low frequency (delta) and high frequency gamma bands were most prominent (Maffei, 2020). As such, band-pass filtering to specific frequency bands is necessary to capture this oscillatory power (Ki et al., 2016). Altogether EEG-ISC analyses can offer complementary information to that observed from fMRI. The previous studies mentioned all used dense electrode systems, either 64 or 128 channels. Although using higher density EEG systems will provide more spatial resolution, a low-density system (14 channel EEG) was also shown sufficient to capture and measure ISC (Poulsen et al., 2017).

Thus, we can use ISC not only to assess how alike subjects are to each other, but also their engagement to the emotional stimuli being presented to them (Poulsen et al., 2017). Several studies have also begun to explore whether ISC differs in individuals with anxiety and depression. For example, ISC is lower in individuals with social anxiety (Morrison et al., 2016), and major depressive disorders (Guo et al., 2015). Electrodermal activity measures (EDA) observed decreased ISC values in dysphoric individuals (Li et al., 2021). Thus, prior work has generally shown that natural videos can be used as a way of identifying “common” patterns of activity that occur across subjects, and individuals with various levels of anxiety and depression may show greater differences from this common pattern of visual-evoked activity, resulting in lower than expected ISC values.

In this study, we wanted to contribute to the above literature in two ways. First, it is increasingly recognized that a larger sample size is required for robust estimates of neurocognitive phenomena (Szucs and Ioannidis, 2020; Feng et al., 2022). As such, we wanted to estimate the degree to which ISC occurs using a large sample of subjects. Second, we were interested in observing, in this large sample, whether ISC might be inversely related to either anxiety or depressive symptoms. To accomplish this, we performed an EEG-based ISC analysis with data parsed into physiologically relevant theta (4–8 Hz), alpha (8–12 Hz), and beta (15–30 Hz) frequency bands as subjects watched an emotionally evocative short movie clip. Additionally, we analyzed the correlation between each subject’s average ISC and mental health symptoms of anxiety and depression. We also use sparse Bayesian source localization to identify brain regions that contribute to the EEG-based ISC.

## Materials and methods

### Participants

A total of 163 human subjects participated in the study (mean age: 39.80 ± 22.65, range: 15–84 years, 59 males). All subjects were fluent in English and provided written informed consent to participate in the study following the University of California San Diego (UCSD) institutional review board (IRB) protocol #180140. Participants were recruited by convenience sampling from the local university and broader San Diego community using flyers and the online Research Match registry. All data collection took place prior to the COVID-19 pandemic research restrictions placed in Spring 2020.

Participants provided demographics data with regards to age, gender, ethnicity, and provided mental health data on standard scales of generalized anxiety (7-item generalized anxiety disorder scale, GAD7) (Spitzer et al., 2006), and depression (9-item patient health questionnaire, PHQ9) (Kroenke et al., 2001). All participants were healthy, i.e., did not have any current medical diagnosis nor were taking any current psychotropic medications.

### Sample size and power

Our participant sample size met criteria for investigating greater than small effect size outcomes across subjects (neurobehavioral correlation coefficient, *r* > 0.1) at beta power of 0.8 and alpha of 0.05, as calculated using the G*Power software (Faul et al., 2009).

### Data acquisition

All participants made individual, single session study-visits at the Neural Engineering and Translational Labs (NEAT Labs) located at the Altman Clinical and Translational Research Institute at the University of California San Diego. Participants logged into the *BrainE* Unity platform (Balasubramani et al., 2021), and viewed the short film “The Lion Cage” by Charlie Chaplin video that lasted 3.83 min. The video was delivered on a Windows-10 laptop at a comfortable viewing distance while recording EEG signals. We did not employ a scrambled video as control because the study goal was focused on investigating effects of anxiety and depression in processing emotional narratives, which requires the stimulus to stay intact. Although a scrambled stimulus maybe beneficial to compare with ISC to real stimuli, previous research has also omitted this control (Dmochowski et al., 2014). The Lab Streaming Layer (LSL) protocol was used to time-stamp the beginning and end of the video clip (Kothe et al., 2019).

Electroencephalography data were collected using a 24-channel saline soaked cap following the 10–20 system and a wireless SMARTING amplifier. The signals were digitized with a sampling rate of 500 Hz and 24-bit resolution and stored as .xdf files.

### Stimulus

“The Lion Cage” depicts a man (Charlie Chaplin) in a circus getting accidentally locked in a cage with a sleeping lion and his efforts to escape without waking the lion. This film was chosen due to its emotional rollercoaster nature (scenes of high stress and anxiety mixed in with emotional relief), which we hypothesized would induce higher emotions in the subjects and show significant levels of ISC. Using a black and white silent film format also reduces confounding variables and puts more focus on the emotional narrative (Vandewalle et al., 2010; Gerdes et al., 2014). The entire film and subsequent data were parsed into 10 consecutive time windows to help isolate scenes of interest, with each window containing an interval of 23 s. This parsing was used for simple standardization (i.e., to have a standardized window of time) and was not related to aspects of the scene or video. The authors also independently coded these 10 windows with varying emotions and intensity scores on a 10-point scale to create an emotion key. After the ratings were proven reliable with a Cronbach’s alpha value of 0.704, the authors met to resolve differences and came to a consensus on a single key for simplicity shown in Table 2 (Cronbach, 1951).

**TABLE 1:** Summary of participant demographics and mental health symptoms self-reported by healthy study subjects.

Demographics	
Age (years, mean ± std)	39.80 ± 22.65
Gender *n* (%)	–
Male	59 (34.4%)
Female	103 (63.6%)
Ethnicity *n* (%)	–
Caucasian	95 (58.6%)
Black/African American	3 (1.9%)
Asian	40 (24.7%)
Native American	2 (1.2%)
More than one ethnicity	14 (8.6%)
Other	8 (4.9%)
Anxiety (mean ± std)	4.11 ± 4.48
Depression (mean ± std)	3.78 ± 4.20

Anxiety and depression were self-reported on the GAD7 and PHQ9 scales, respectively.

**TABLE 2:** Description of the 10 scene segments and potential evoked emotional responses as keyed by the authors.

Window # (Seconds)	Scene	Evoked response
1 (0–23)	Charlie Chaplin (CC) is in a cage with a sleeping lion	C(6.3), F(6)
2 (23–46)	CC waves a handkerchief to try and get help	S(6)
3 (46–69)	CC crawls through a hole where he is unknowingly close to a tiger	F(7), A(6)
4 (69–92)	There is a dog jumping and barking at the cage with the sleeping lion	S(4.6), AG(4.3), F(3), H(3)
5 (92–115)	A woman approaches the cage and subsequently passes out	AG(4), S(3), H(3),
6 (115–138)	The lion wakes up and approaches CC	F(8)
7 (138–161)	The lion walks away, rolls over onto his back, and goes to sleep	R(5.3), H(3.7), S(3)
8 (161–184)	The woman wakes up and opens the cage door; CC showcases a sense of relief	R(8.7)
9 (184–207)	CC runs out of the cage and up a flagpole to get away from the lion’s cage	H(7.3), F(3.3), S(3), A(3)
10 (207–230)	Film ends with CC taking a bow	R(6), H(5.3)

Emotion key: F, fear; A, anxiety; S, Stress; R, relief; AG, anger; C, confusion; H, humor. The number in parentheses is the average intensity score given by the authors on a 10 point scale (Cronbach’s alpha = 0.704).

### Pre-processing of electroencephalography channel data

A standard pipeline was used for all subjects to clean the data through EEGLAB v 14.1.2 in MATLAB (Delorme and Makeig, 2004). Using EEGLAB, the data was resampled to 250 Hz and bandpass filtered between 1 and 45 Hz. This was achieved with the eegfiltnew() function which uses an 826 order Hamming windowed sinc FIR filter (Widmann et al., 2015). This removes DC drift at <1 Hz and high frequency noise originating from muscle contractions or 60 Hz line noise. All channels used an average reference. The EEG data were then parsed to isolate the start and stop of the film while also removing any pauses during the film’s playback from event markers generated by the LSL. Artifact rejection was performed automatically using the Sparse Bayesian Learning Algorithm (BSBL) (Ojeda et al., 2018, 2021) to remove non-EEG-signals, i.e., signals of electrooculographic (EOG), electromyographic (EMG), or unknown source origin. Outlier rejection was done to further clean the data, excluding any data points that were greater than 5 standard deviations (5SD) above the average. The cleaned data was then filtered into three frequency bands, theta (4–8 Hz), alpha (8–12 Hz), and beta (15–30 Hz) for individual analysis. Our experimental setup was not in a very low-noise/shielded environment to allow for gamma band analyses, hence, this frequency band was not included in the analyses.

### Inter-subject correlation

To extract the instantaneous power for correlation analysis, we transformed each subject’s channel data into Hilbert space, calculated the magnitude at each discrete time point (Freeman, 2004) and then standardized the data by *z*-scoring. Using the MATLAB function glmfit, we fit linear regression models for every pair of subjects using the 23 s of data within each of the time windows across the 24 electrodes and three frequency bands. Thus, for each electrode, time-window and frequency band we created a symmetric N × N matrix (β) where N is the number of subjects and β_*ij*_ represents the beta value (slope) between subjects i and j. In other words, the β matrix is comprised of individual beta values for all pairwise combinations of subjects. These beta values are regression coefficients and thus not scaled between ±1. *P*-values were calculated from the upper triangular elements of this matrix using a one-sample *t*-test across all unique intersubject β_*ij*_ values for significance relative to the null hypothesis, with the null hypothesis being that the average inter-subject beta value would be 0 for all electrode, time window and frequency combinations indicating no correlation between any of the subjects (Wilson et al., 2008; Kauppi, 2010). A total of 100 iterations of permutation testing were also performed for the peak window to estimate the likelihood of mean beta-values above occurring from uncorrelated data. Each subject’s neural activity was scrambled across time, followed by the ISC calculation as described above for each frequency and electrode. These 100 repeats served as our random distribution, and we calculated the percentile at which the real data sat relative to the random distribution. Permutation testing confirmed significance of the real beta values in the peak window at greater than 99th percentile of the permuted random distribution.

Average beta values for each electrode and time window were plotted on a heatmap masked with significance. Adjusted *p*-values from Benjamini-Hochberg false discover rate (FDR) were used to resolve multiple comparisons across the 24 electrodes and 10 time windows. We identified the 8th time window (at 184–207 s in the video) for further analysis as it showed the largest and most significant ISC values when averaged across electrodes for each frequency band. This was further confirmed by running a repeated measures ANOVA across the 10 time windows followed by Tukey’s *post-hoc* test. Beta values were averaged across frequency and electrodes to find the overall peak window of interest. We chose to analyze the peak ISC because that would presumably be the timeframe of highest engagement across subjects as well as when healthy subjects are most “in sync” (Dmochowski et al., 2012; Cohen et al., 2017, 2018). The ISC data for this time window was then plotted on a 2D scalp topography representation for better visualization with the topoplot.m function in MATLAB’s EEGLAB toolbox. In addition to the ISC data, the average activity across subjects during the peak ISC window, i.e., 8th time window, was plotted in a similar manner to determine peak activity electrode clusters for further analysis. The topography plots revealed the midline centro-parietal region electrodes (Cz, CPz, Pz, POz) had highest ISC values as well as neural activity. Peak ISC and neural activity were compared across frequency bands using within-subjects repeated measures analysis of variance (rm-ANOVA) with the Greenhouse–Geisser correction applied to adjust for lack of sphericity.

### Source-localized analysis

We performed cortical source localization to map the underlying neural source activations using the block-sparse Bayesian learning (BSBL-2S) algorithm (Ojeda et al., 2018, 2021). This is a two-step algorithm in which the first-step is equivalent to low-resolution electromagnetic tomography (LORETA) (Pascual-Marqui et al., 1994). LORETA estimates sources subject to smoothness constraints, i.e., nearby sources tend to be co-activated, which may produce source estimates with a high number of false positives that are not biologically plausible. To guard against this, BSBL-2S applies sparsity constraints in the second step wherein blocks of irrelevant sources are pruned. This data-driven sparsity constraint of the Sparse Bayesian Learning (SBL) method reduces the effective number of sources considered at any given time as a solution, thereby reducing the ill-posed nature of the inverse mapping (Ojeda et al., 2018, 2021). In other words, one can either increase the number of channels used to solve the ill-posed inverse problem or impose more aggressive constraints on the solution to converge on the source model when channel density is low/moderate; 24 channels in this case. The two-stage SBL has been benchmarked to produce evidence-optimized inverse source models at 0.95 AUC relative to the ground truth, while without the second stage < 0.9 AUC is obtained (Ojeda et al., 2018, 2021). Prior research also provides support that sparse source imaging constraints can be soundly applied to low channel density data (Ding and He, 2008; Stopczynski et al., 2014), and we have also shown that cortical source mapping with this method has high test-retest reliability with Cronbach’s alpha of 0.77 (Balasubramani et al., 2021).

Prior to source analysis, EEG data were specifically filtered in theta (4–8 Hz), alpha (8–12 Hz), and beta (15–30 Hz) bands and separately source localized in each of the three frequency bands to estimate their cortical ROI source signals. Using BSBL-2S, the 24-channel frequency band specific data were mapped onto 68 cortical brain regions as defined by the Desikan-Killiany (DK) atlas (Desikan et al., 2006) with the Colin-27 head model (Holmes et al., 1998). For this, the source model included 8,003 dipoles that were then parcellated into the DK atlas 68 cortical regions by averaging the magnitudes of the dipole sources in the same cortical region (Ojeda et al., 2018, 2021). The signal envelope was calculated to obtain the source spectral amplitude of each brain region across time in each subject. Artifacts from external factors such as jaw or eye movement that may remain in source space require an alternate approach for outlier rejection beyond 5 SD; for these, we employed the Grubbs statistical test to iteratively remove outliers and replace them using a spline interpolation—an option available within the MATLAB isoutlier function. ISC was performed on the cleaned subject source data in all ROIs and the average ISC across subjects in peak time window 8 was plotted on cortical brain maps in the three frequency bands with one-sample *t*-test significance testing of β coefficients performed relative to null; *p*-values were FDR corrected across 68 brain regions and three frequency bands.

### Neurobehavioral correlations with mental health

We used Spearman’s correlation to investigate associations between each subjects’ anxiety (GAD7)/depression (PHQ9) score and their average ISC value within the peak window and electrode cluster identified earlier. Spearman’s correlation analyses were used as these are less sensitive (though not completely insensitive) to outlier effects (Rousselet and Pernet, 2012). Results were FDR *p* < 0.05 corrected for multiple comparisons. Spearman’s rho (ρ) values indicated effect size: 0.1: small, 0.3: medium, 0.5: large effect size. Age and gender were also tested as covariates using Spearman’s partial correlations.

## Results

Table 1 shows subject demographic and mental health (anxiety and depression) symptom report data that was available for 162 of 163 human subject participants. The sample had about a 2:1 ratio of females to males and a Caucasian majority. Subjects with anxiety/depression scores > 5 are considered to have mild symptoms; there were 28.75 and 22.01% subjects with mild anxiety and mild depression symptoms, respectively.

**FIGURE 1:**
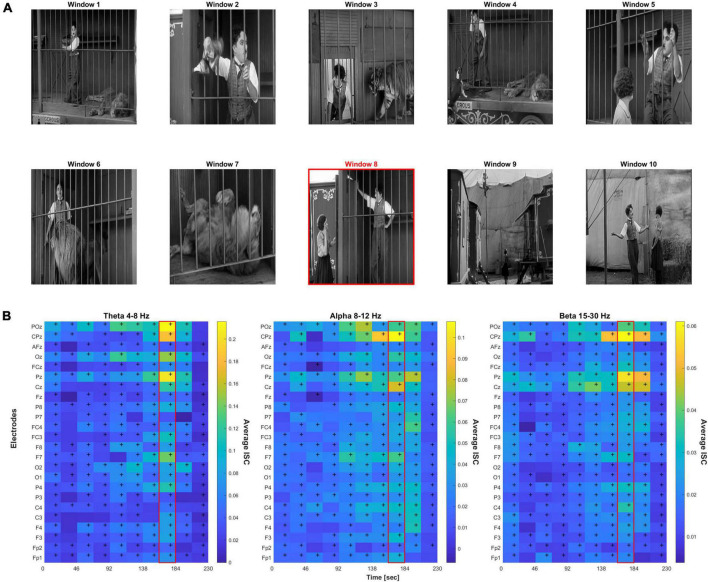
**(A)** Screenshots representative of 10 time windows from the film. The red border indicates the peak 8th time window. **(B)** Heat map of the inter-subject correlation (ISC) values per electrode and time window across three frequency bands; peak ISC was observed in the 8th time window. Cells with “+” indicate FDR-corrected *p* < 0.05 significance relative to null.

Figure 1 shows brief screenshots of the short film where each screenshot is meant to capture the scene of each particular 23 s time window. Below it are heatmaps of the ISC values across the film’s 10 time windows, electrodes, and frequency bands. Repeated measures ANOVA resulted in *F*(9,1.2E5) = 32.9, *p* < 1.3E-36 after Greenhouse–Geisser correlation for sphericity. *Post-hoc* Tukey’s test revealed that peak window 8 was significantly different than all other windows (*p* < 0.0002). Table 2 provides a scene description by time window, and the potential emotion that may be evoked when watching the scene.

From the ISC heatmaps, we observed that highest average inter-subject correlation across electrodes was found during time window 8 (highlighted in red in Figure 1 scene panel), which coincided with the permanent onset of emotional relief. Almost all electrodes showed significant correlation across time, *p* < 0.05 FDR-corrected. FDR-correction was performed across the 24 electrodes and 10 time windows, i.e., correcting for 240 statistical tests, separately for each frequency band. For all further analyses, we focused on the peak ISC time window 8 which had the highest relief rating by all authors across the 10 scenes.

**FIGURE 2:**
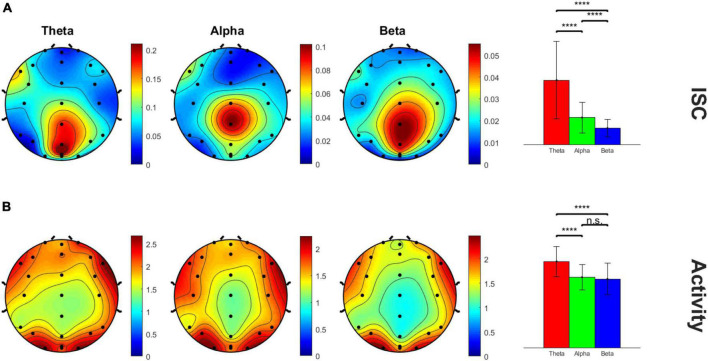
Scalp topography maps of peak ISC window 8 across three frequency bands. **(A)** ISC values and **(B)** average neural activity on the scalp. The bar graph panel on the right plots the mean ± std peak time window ISC and neural activity values at midline centro-parietal electrodes across the three frequency bands. ****p < 0.001.

Figure 2 shows the scalp topography of the peak time window 8 ISC, showing clear focality in the midline centro-parietal electrodes in all theta, alpha and beta frequency bands. The B panel shows the corresponding average neural activity during the same time window, which interestingly showed lateral but not central maxima, suggesting ISC magnitude was not a simple function of activity magnitude. The theta frequency band appeared by eye to have the highest magnitude ISC values compared to the other frequency bands; hence, we statistically analyzed this. The bar graphs in the right panel quantify and compare the magnitude of ISC (A) and activity (B) across frequency bands in the peak (Cz, CPz, Pz, and POz) electrodes (within-subjects rm-ANOVA across frequency bands, ISC: *F*(2, 46) = 45.42, *p* = 1.95E-7, average activity: *F*(2, 46) = 22.52, *p* = 4.1E-5). ISC was largest for the theta frequency band compared to both other frequencies, and alpha showed higher ISC than beta frequency bands.

**FIGURE 3:**
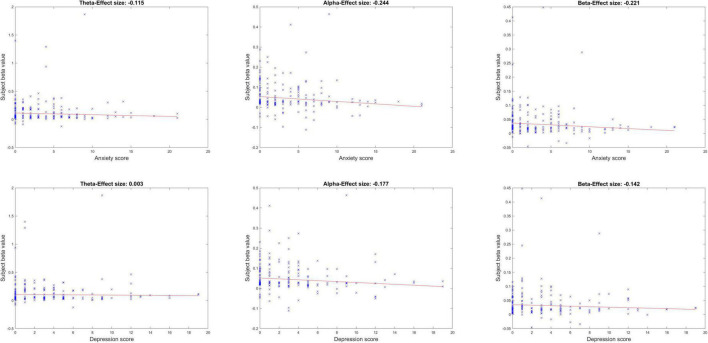
Neurobehavioral correlations between peak ISC and mental health symptoms of anxiety and depression, across the three frequency bands.

Figure 3 shows the neurobehavioral correlations between the peak ISC (within peak time window eight and peak midline centro-parietal channels) and subjects’ mental health scores. All correlations were FDR-corrected for multiple comparisons. Neurobehavioral correlations with anxiety in the alpha and beta band showed a significant inverse relationship [alpha band rho (ρ) = –0.244, *p* = 0.012; beta band ρ = –0.221, *p* = 0.015]; correlation with anxiety in the theta band was not significant (theta band ρ = –0.115, *p* = 0.179). Neurobehavioral correlations were anxiety specific and did not achieve FDR-corrected significance with depression scores in our healthy sample (*p* > 0.05). Age and gender were not significant covariates of these ISC-anxiety neurobehavioral relationships (Spearman partial correlations, alpha ISC vs. age ρ = –0.073, *p* = 0.361; vs. gender ρ = –0.123, *p* = 0.123; beta ISC vs. age ρ = 0.017, *p* = 0.831; vs. gender ρ = –0.129, *p* = 0.105; although age and anxiety were significantly inversely related in these partial correlations: ρ = –0.33, *p* < 0.0001, anxiety and gender were not: ρ = 0.08, *p* = 0.3).

**FIGURE 4:**
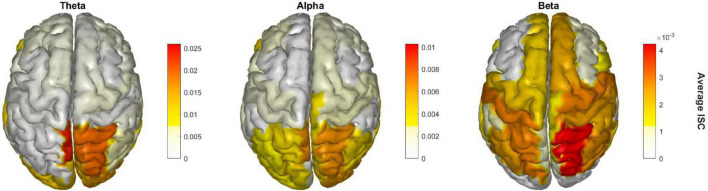
Significant brain regions of ISC during the peak time window across the three frequency bands.

Finally, Figure 4 shows the EEG cortical source reconstruction of the peak window ISC data, mapped onto the 68 ROI brain regions as per the Desikan-Killiany atlas. Similar to Figure 3, theta band ISC had larger magnitude values than alpha and beta band with greatest intensity in the right superior parietal cortex across all bands.

## Discussion

Inter-subject correlation of physiological data can provide useful proof of how similarly humans process stimulus information (Hasson et al., 2004). Several neuroimaging studies using fMRI during video watching have shown evidence of ISC in sensory, multisensory and limbic brain regions (Jääskeläinen et al., 2008; Nummenmaa et al., 2012; Di and Biswal, 2020). Recent studies have utilized EEG for generating ISC during classroom video watching (Poulsen et al., 2017) and to detect stress/anxiety states from EEG features during video watching (Giannakakis et al., 2015). Here, we extend this literature to ask whether ISC during naturalistic video watching especially in a large subject sample relates to mental health. We find that ISC, as a physiological marker of inter-subject neural processing similarity during passive viewing, has a significant negative relationship with anxiety, showing stronger correlation in subjects without anxiety than with anxiety.

We observed that peak ISC was obtained in EEG signals when passively viewing a particular scene segment (161–184 s) in a video clip of Charlie Chaplin’s (CC) film “Lion Cage” that was 230 s in total duration. This 8th time window is observed to coincide with the possible onset of strongest emotional relief in the film when CC is let out of the dangerous lion’s cage. This period contrasts to the rest of the film up to this point, potentially transitioning viewers to a more relaxed state of mind. As previous research has suggested, we would expect to see high ISC with low variation across subjects as they all go through the same emotional response (Jääskeläinen et al., 2008). Interestingly, the magnitude of peak ISC in the alpha/beta frequency bands at the centro-parietal electrodes was inversely related to subjects’ self-reported anxiety, i.e., more anxious individuals reporting greater anxiety had lower ISC scores. Greater activity in the alpha and beta bands have been related to anxiety and stress (Knyazev et al., 2004; Price and Budzynshi, 2009; Abhang et al., 2016). More pertinently, it is possible that individuals with higher levels of anxiety are less distinct during this epoch when, in general, there is a sense of “relief.” Higher theta activity is also expected with the presentation of visual stimuli as well as high facial perception, which is also observed in our analysis (Busch et al., 2009; Dravida et al., 2019). We further verified that these neurobehavioral correlations were not affected by age or gender. We also confirmed that this peak ISC localized to the superior parietal brain region, which is a well-known component of the attentional network (Corbetta and Shulman, 2002; Dosenbach et al., 2007). Meta-analyses have identified this region as important for attention and emotion, coding for emotional dysregulation especially in mood and anxiety disorders (Compton et al., 2003; Picó-Pérez et al., 2017). ISC-anxiety correlations being most significant in the alpha and beta bands suggests local region-specific modulation selective for attention and emotion processing (Ray and Cole, 1985; Klimesch et al., 1998; Schutter et al., 2001; Güntekin and Basar, 2007; Güntekin and Başar, 2010; Peylo et al., 2021). Alpha activity in parietal cortex has been postulated to select visual information for attentive processing (Peylo et al., 2021), and the parietal alpha ISC-anxiety correlations may suggest that individuals with greater anxiety may not process relief, the positive emotion in the peak time window, as readily as those with lower anxiety scores.

Limitations of this research include use of a moderate density electrode system, which may lead to more approximate source localization; these results could be verified by high density EEG mapping or other neuroimaging in future research. Furthermore, the lack of having multiple videos (and/or scrambled videos) as a control stimulus, may complicate the interpretation of our results. With regards to neural signal processing, we used a bandpass filtering approach to extract frequency band information. However, as noted in Widmann et al. (2015), separate low and high pass filtering may be better for attenuating the signals below the cutoff frequency particularly for the high-pass filters. Thus, it is possible that our data within theta, alpha and beta frequencies have some degree of contamination from outside the filter band. This contamination effect may to some extent explain why we observe similar findings in the alpha and beta frequencies, in particular, similar correlations with anxiety. Importantly, though, the theta and alpha frequency bands are both narrower and closer together and thus more likely to show cross-band contamination, and we found that, while alpha-frequency electrodes significantly correlated with symptoms such as for depression (*r* = –0.177), theta frequency band signals did not (*r* = 0.003), suggesting some frequency band specificity. An additional limitation of this study is that our symptom correlation analysis relied purely on subjective reporting on symptoms scales of anxiety and depression. We did not have a systematic way to interrogate or otherwise clinically verify whether these scores were an accurate depiction of an individual’s actual level of anxiety or depression, although notably the prevalence of symptoms in this study reflects the prevalence of anxiety/depression in a mixed community and college sample (Kessler et al., 2012; Li et al., 2022). That symptom scales may have been misreported, or there may simply have been some misunderstanding as individuals were responding, is a small possibility. However, we believe this type of misreporting/error would have been more likely to reduce rather than increase any neurobehavioral correlations observed, and is a general challenge with many neurobehavioral correlation studies. As such, results also need to be extended to individuals with clinically diagnosed anxiety to investigate if these relationships hold true in more anxious individuals in a clinical population.

In conclusion, the neurophysiological ISC measures may parallel the mirrored human emotions elicited during video watching. Being able to properly understand and feel similar emotions as others around us, i.e., empathy, is an important trait possessed by healthy humans. Yet, research has shown that individuals with anxiety, especially social anxiety, have difficulty understanding positive emotions of others as readily as healthy controls (Morrison et al., 2016). Other research suggests that empathy may be intact in social anxiety but that there may be deficits in prosocial action (Auyeung and Alden, 2016, 2020), and that links between anxiety and empathy are not fully clear (Pittelkow et al., 2021). Our physiological analysis suggests that aspects of parietal attention, especially during a period of positive emotion, i.e., relief from prior stress during emotional video viewing, may not occur as readily in more anxious individuals. Furthermore, we show that these neurobehavioral relationships hold true for continuous data that is not event-locked to specific stimuli.

## Data availability statement

The raw data supporting the conclusions of this article will be made available by the authors, without undue reservation.

## Ethics statement

The studies involving human participants were reviewed and approved by the University of California San Diego IRB. The patients/participants provided their written informed consent to participate in this study.

## Author contributions

PB, DR, and JM contributed to the conception and design of the study. JN performed the statistical analysis and wrote the first draft of the manuscript. All authors contributed to the manuscript revision, read, and approved the submitted version.
